# Insights into postoperative respiration by using continuous wireless monitoring of respiratory rate on the postoperative ward: a cohort study

**DOI:** 10.1007/s10877-019-00419-4

**Published:** 2019-11-13

**Authors:** Linda M. Posthuma, Maarten J. Visscher, Philipp B. Lirk, Els J. M. Nieveen van Dijkum, Markus W. Hollmann, Benedikt Preckel

**Affiliations:** 1 Department of Anesthesiology, Amsterdam UMC, University of Amsterdam Academic Medical Centre AMC, Meibergdreef 9, P.O. Box 22660, 1100 DD Amsterdam, The Netherlands; 2 Department of Anesthesiology, Brigham and Women’s Hospital, Harvard Medical School, 75 Francis Street, Boston, MA 02115 USA; 3 Department of Surgery, Amsterdam UMC, University of Amsterdam Academic Medical Centre AMC, P.O. Box 22660, 1100 DD Amsterdam, The Netherlands

**Keywords:** Monitoring, Patient safety, Respiratory rate, Vital parameter, Wireless monitoring

## Abstract

Change of respiratory rate (RespR) is the most powerful predictor of clinical deterioration. Brady- (RespR ≤ 8) and tachypnea (RespR ≥ 31) are associated with serious adverse events. Simultaneously, RespR is the least accurately measured vital parameter. We investigated the feasibility of continuously measuring RespR on the ward using wireless monitoring equipment, without impeding mobilization. Continuous monitoring of vital parameters using a wireless SensiumVitals® patch was installed and RespR was measured every 2 mins. We defined feasibility of adequate RespR monitoring if the system reports valid RespR measurements in at least 50% of time-points in more than 80% of patients during day- and night-time, respectively. Data from 119 patients were analysed. The patch detected in 171,151 of 227,587 measurements valid data for RespR (75.2%). During postoperative day and night four, the system still registered 68% and 78% valid measurements, respectively. 88% of the patients had more than 67% of valid RespR measurements. The RespR’s most frequently measured were 13–15; median RespR was 15 (mean 16, 25th- and 75th percentile 13 and 19). No serious complications or side effects were observed. We successfully measured electronically RespR on a surgical ward in postoperative patients continuously for up to 4 days post-operatively using a wireless monitoring system. While previous studies mentioned a digit preference of 18–22 for RespR, the most frequently measured RespR were 13–16. However, in the present study we did not validate the measurements against a reference method. Rather, we attempted to demonstrate the feasibility of achieving continuous wireless measurement in patients on surgical postoperative wards. As the technology used is based on impedance pneumography, obstructive apnoea might have been missed, namely in those patients receiving opioids post-operatively.

## Introduction

Monitoring of vital parameters including blood pressure, heart rate, respiratory rate (RespR, measured as breath per minute), peripheral oxygen saturation, urine output and temperature is a key measure contributing to the modified early warning scores (MEWS) for early detection of patient deterioration, thereby preventing “failure-to-rescue” events [1]. In most post-operative wards, these vital parameters are measured intermittently (typically every few hours) during a nurse shift, thus about three times a day. In more instable patients, these measurements might be performed more frequently, but time between observations is often longer than 4 h [2]. Frequency of vital signs measurement has increased after implementation of the MEWS [3], however patient deterioration in-between measurements can still go unnoticed [4].

When only one vital parameter is used to monitor patients, alterations in RespR are the most powerful predictor to detect clinical deterioration [5, 6]. Respiratory depression plays a significant role in post-surgical patients treated for pain with opiods [7]. During recent years, wireless monitoring systems became available [8], allowing for continuous measurement of RespR outside high-care wards. However, previous studies with these emerging technologies measured RespR just for a very short period of time [9–11], or the respective wireless system lacked accuracy [12]. Most previous studies focussed on medical patients [13–15]. However, postoperative patients are exposed to circumstances directly influencing RespR, e.g., surgery itself, anaesthesia as well as postoperative pain treatment, and the insidious occurrence of complications such as infection and sepsis [16–18].

While standardised RespR measurement show a normal distribution [13], documentation of RespR in clinical routine show a bias towards digits as 18, 20 or 22 [19, 20]. Measurement of RespR has historically occurred inconstantly [3], often inaccurately [19, 21] and was frequently guessed, estimated or just repeated in the record from previous measurements [20]. Need for proper training of nurses for taking vital signs has been emphasised before [22].

In the present study we determined the feasibility—defined as at least 50% of technical valid measurements resulting in at least 360 measurements of RespR per day in more than 80% of patients—of continuous wireless monitoring for up to 4 days post-operatively on the post-surgical ward in the mobilizing patient. In addition, we hypothesised that in the post-surgical patient population RespR still will be normally distributed, with mean values of RespR lower than the previously reported 20 breaths per minute. For measuring RespR, a Sensium Vitals® patch was applied via standard electrocardiogram electrodes to the chest of post-surgical patients. The patch measures heart rate, RespR (based on impedance pneumography) and axillary temperature.

## Methods

### Measurement device and data collection

**Fig. 1:**
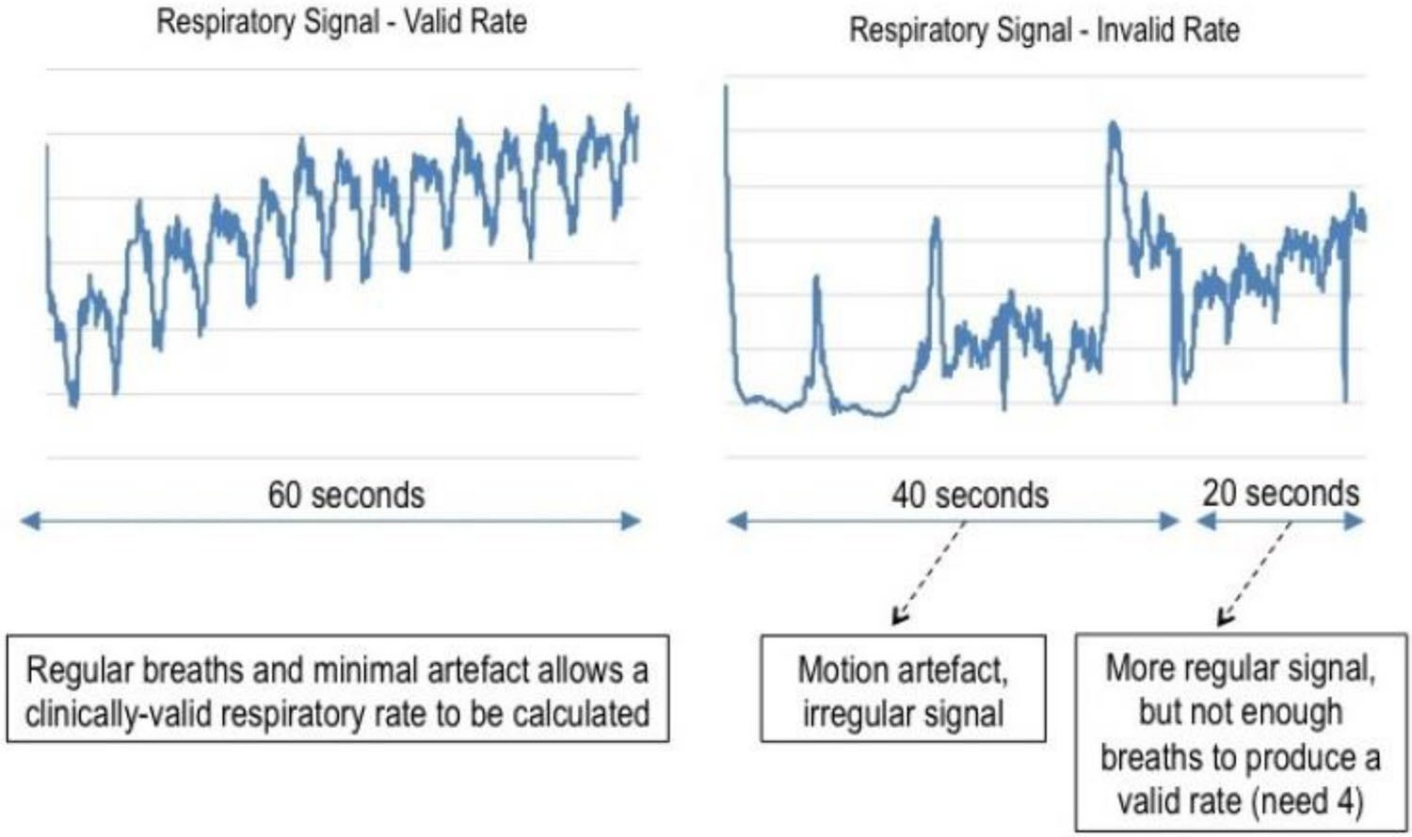
Examples for valid and invalid measurements of respiratory rate

For this prospective observational study we technically installed a continuous wireless monitoring system (SensiumVitals®) on two wards serving post-operative patients mainly after gastrointestinal surgery [9, 13, 23, 24]. Only non-sedated, not ventilated patients were included. Patients were equipped with a wireless SensiumVitals® patch as per manufacturer's instructions (Sensium Healthcare, London, UK) either in the recovery area or on the post-operative ward. This patch is fixed in the middle of the chest using two standard electrocardiogram electrodes, and standard medical tape to fix the temperature sensor in the axilla [9, 24], and measures RespR, heart rate and temperature every 2 mins. After activation by removing a plastic lit, the patch measures heart rate by an electrocardiogram segment for 30 s, followed by measuring RespR for 60 s by impedance pneumography. An algorithm in the patch starts with a conditioning stage, filtering the raw respiration waveform to minimize noise due to heart activity and motion artifacts. This stage is followed by a detection stage that applies a number of thresholds and a set of heuristic and physiological rules, to detect and discriminate real breathing signals from spurious/corrupted ones. Once the respiration cycle is completed, the algorithm either calculates the average RespR, or it rejects the signal as invalid due to excessive contamination by noise (invalid data). These invalid data will not be used for alarm/notification generation, and therefore will not lead to false alarms, thus not increasing nurses’ workload. Valid data of the patch are sent to a radiofrequency identification (RFID) bridge installed throughout the surgical ward, from where the measured data are sent to a server. When data acquisition is interrupted by motion and/or electric irregularities, an internal algorithm within the patch will detect these erroneous measurements and will automatically reject them before sending the data to the bridge. In these respective cases, instead of data for RespR an error message (so-called invalid measurement, see Fig. 1) is send to the bridge. Error messages are forwarded, allowing ward clinicians to see whether a patient is technically well monitored or not. Accuracy of the system to adequately measure RespR during surgery and for 2 h on the ward was demonstrated before [9]. The system reported RespR measures 50% of time (called valid measurements) [9].

Based on previous studies we defined feasibility of adequate RespR monitoring during a prolonged observation period in the mobilizing post-surgical patient if the system reports RespR measurements (so-called valid measurements) in at least 50% of time-points in more than 80% of patients during day- and night-time, respectively [9]. This would result in a mean of at least 15 out of 30 RespR measurements per hour. As secondary outcome we measured the average RespR in postoperative patients, expecting a normal distribution. Critical changes in RespR, e.g., brady- and tachypnea, were determined. Bradypnea was defined as a RespR ≤ 8; tachypnea was defined as a RespR ≥ 21 [Systemic Inflammatory Response Syndrome (SIRS) criteria] or above RespR ≥ 31 (according to the local MEWS score).

### Study population and sample size estimation

Hernandez et al. measured vital parameters for 2 h in 25 patients lying in bed, but recommended the use of ‘significant sample of participants in the general ward’ for further studies [9]. To account for invalid measurements due to mobilizing patients, we intended to include at least 100 post-surgical mobilizing patients. During the summer period, around 60 eligible patients per months on the respective wards underwent surgery. Therefore, all eligible adult patients undergoing moderate and major general surgery in June, July and August 2017 of the respective wards were enrolled in this prospective observational pilot study in the Amsterdam University Medical Centers, location Academic Medical Center AMC, Amsterdam, the Netherlands. Patients were included if they stayed at least one night on the post-surgical ward. Patients were excluded if they had a pacemaker, were younger than 18 years old, or were sent to the intensive care unit postoperatively. Wearing a patch did not interfere with the normal workflow of patients (e.g., undergoing diagnostic procedures, mobilizing, showering etc.). The patch did not affect patient´s care, and all routine medical and nursing protocols were followed. We did not use a reference monitor to determine RespR and did not act on any deviating measurements. Nursing staff had no access to the measurements. The Medical Ethical Committee of the AMC waived approval for this observational study (W217_205#17.236); informed consent from the patients for analysing the recorded data from the monitoring equipment was obtained and documented.

### Data analyses and statistics

All data on RespR were collected in a database starting with postoperative application of the patch until the end of the fourth post-operative night, or until hospital discharge, whichever came first. Data were than analysed for specific time frames, e.g., day- versus night-time, with day-time running from 8:00 to 20:00 h and night-time from 20:00 to 8:00 h. Because time of data acquisition on the first day mainly depended on scheduling of the operation, we determined the respective vital data on the first day, but started analysis of data divided into day- and night-time from start of night one. Before analysing the data, raw data of the 2.5% highest and lowest values were manually checked, and in case unreliable data were found these values were rejected. Descriptive statistics were used and data are presented as median (interquartile range), mean (95% confidence interval) or percentage.

## Results

One hundred and twenty-six patients were eligible for enrolment in this study. Five patients were admitted from the operating room to the intensive care unit post-operatively, and two patients denied informed consent to analyse their recorded data. Thus, data from 119 patients were analysed. Patients wore the patch for an average time of 64 h (range 4–96 h). Patient characteristics and risk of surgical procedures (minor, intermediate, high risk procedure) are summarised in Table 1.

**Table 1:** Patient characteristics, surgical procedures, type of anaesthesia and postoperative pain management

Patient characteristics	N = 119
Female gender, n (%)	62 (52.1)
Age mean, years (range)	64.7 (17–88)
Body mass index (BMI; kg/m^2^)—median (range)	25.4 (15.0–37.6)
ASA score, n (%)	
ASA 1	11 (9.2)
ASA 2	74 (62.2)
ASA 3	32 (26.9)
ASA 4	2 (1.7)
Comorbidities	
COPD	10 (8.4)
OSAS	0 (0)
Asthma bronchiale	6 (5)
Type op surgery n (%)	
Minor surgery	36 (30)
Intermediate surgery	34 (29)
Major surgery	49 (41)
Type of anaesthesia	
General anaesthesia	89 (74.8)
General anaesthesia with epidural analgesia	30 (25.2)
Pain medication on ward	
Epidural analgesia	26 (21.8)
Wound catheter	14 (11.8)
PCA morphine	56 (47.1)
PCA buprenorphine	7 (5.9)
Oral opioids	70 (59)
Transcutaneous opioid	4 (3.4)

Type of surgery: following cardiac risk stratification for non-cardiac surgery, minor surgery, cardiac risk < 1%; intermediate surgery, cardiac risk 1–5%; major surgery, cardiac risk > 5%

*ASA* American Society of Anesthesiologists, *COPD* chronic obstructive pulmonary disease, *OSAS* obstructive sleep apnoea syndrome, *PCA* patient controlled analgesia

### Valid or invalid measurements

The algorithm within the patch detected in 171,151 of 227,587 measurements valid data for RespR (75.2%). At postoperative day and night four, the system still registered 68% and 78% valid measurements, respectively. During all time frames, the system measured at least 67% valid RespR’s (range 67–81%), demonstrating feasibility (defined as at least 50% of technical valid measurements) of the system to measure RespR up to 4 days postoperatively. Table 2 shows valid versus invalid data per time frame. There were less data rejected (“invalid measurements”) during night-time (20%) than during day-time (33%).

**Table 2:** Valid and invalid RespR measurements per time frame (as % of total measurements)

	Day 1	Night 1	Day 2	Night 2	Day 3	Night 3	Day 4	Night 4
Valid (%)	67.4	81.3	67.1	79.7	67.3	79.2	68.2	78.3
Invalid (%)	32.6	18.7	32.9	20.3	32.7	20.8	31.8	21.7

Ninety patients (88% of included patients) had more than 67% of valid RespR measurements, 11 patients (9%) had even more than 90% valid RespR measurements. In only nine patients (8% of included patients) the signal was rejected in more than 50% of measurements. There were two patients with less than 33% of valid measurements (28% and 20%, respectively). We could not elaborate a reason for this malfunction. The longest period in which no valid measurement was registered was 142 min.

### Respiratory rate

**Fig. 2:**
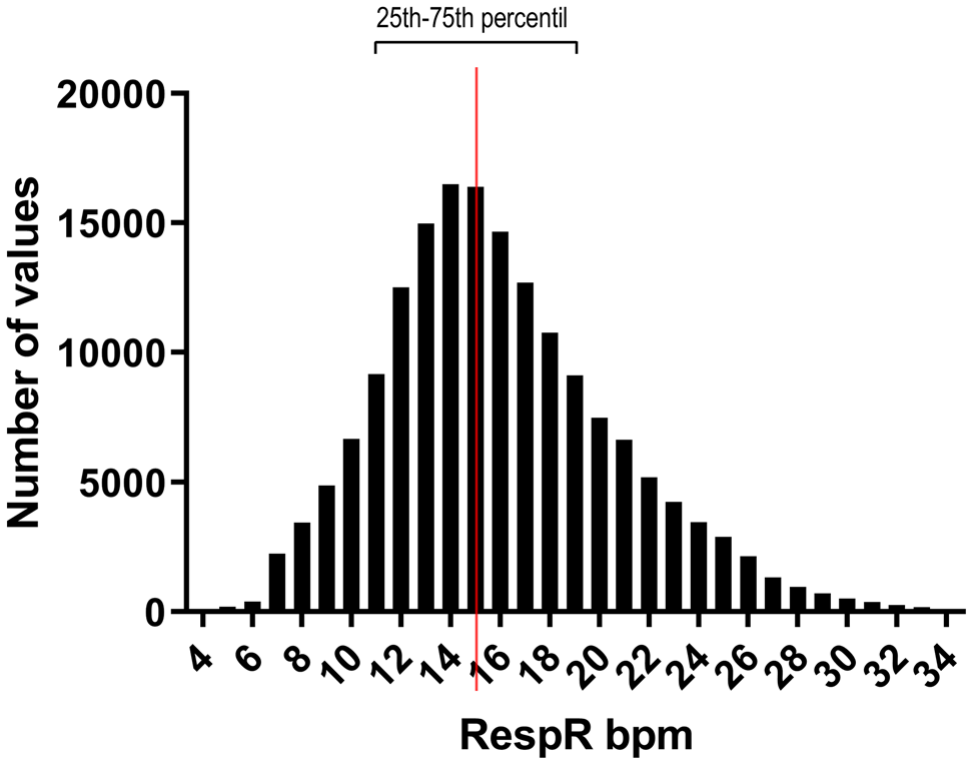
Overall distribution of respiratory rates measured post-operatively on the ward until the end of 4th post-operative night

The RespR’s most frequently measured were 13 (14,978 times), 14 (16,496 times), 15 (16,384 times), and 16 (14,655 times); the median RespR value is 15 (mean 16.0), the 25th- and 75th percentile are 13 and 19, respectively (see Fig. 2). The data were not normally distributed and skewed to the right.

**Fig. 3:**
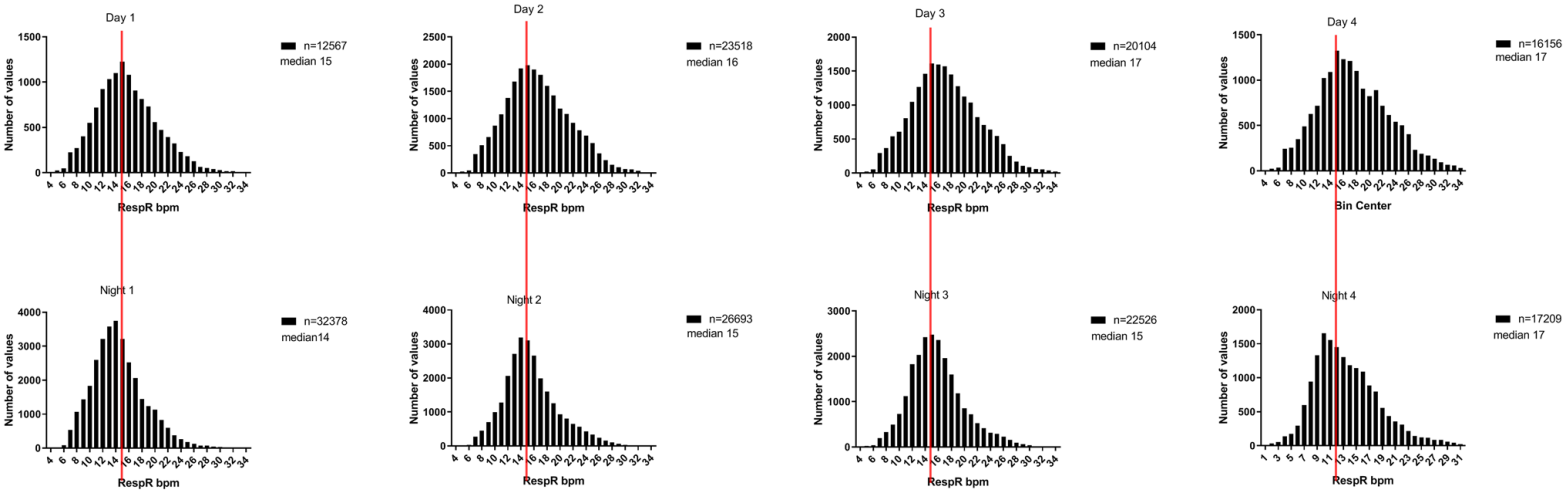
Distribution of respiratory rates measured on the ward during day-time and night-time (red line indicates general median value of 15)

Median RespR during day-time was higher than during night-time, and RespR were more broadly distributed during the day than during the night (see Fig. 3). A slight increase in RespR during the later post-operative days was observed (median post-operative night 1: 14, median postoperative night 4: 17).

### Bradypnea and tachypnea

**Fig. 4:**
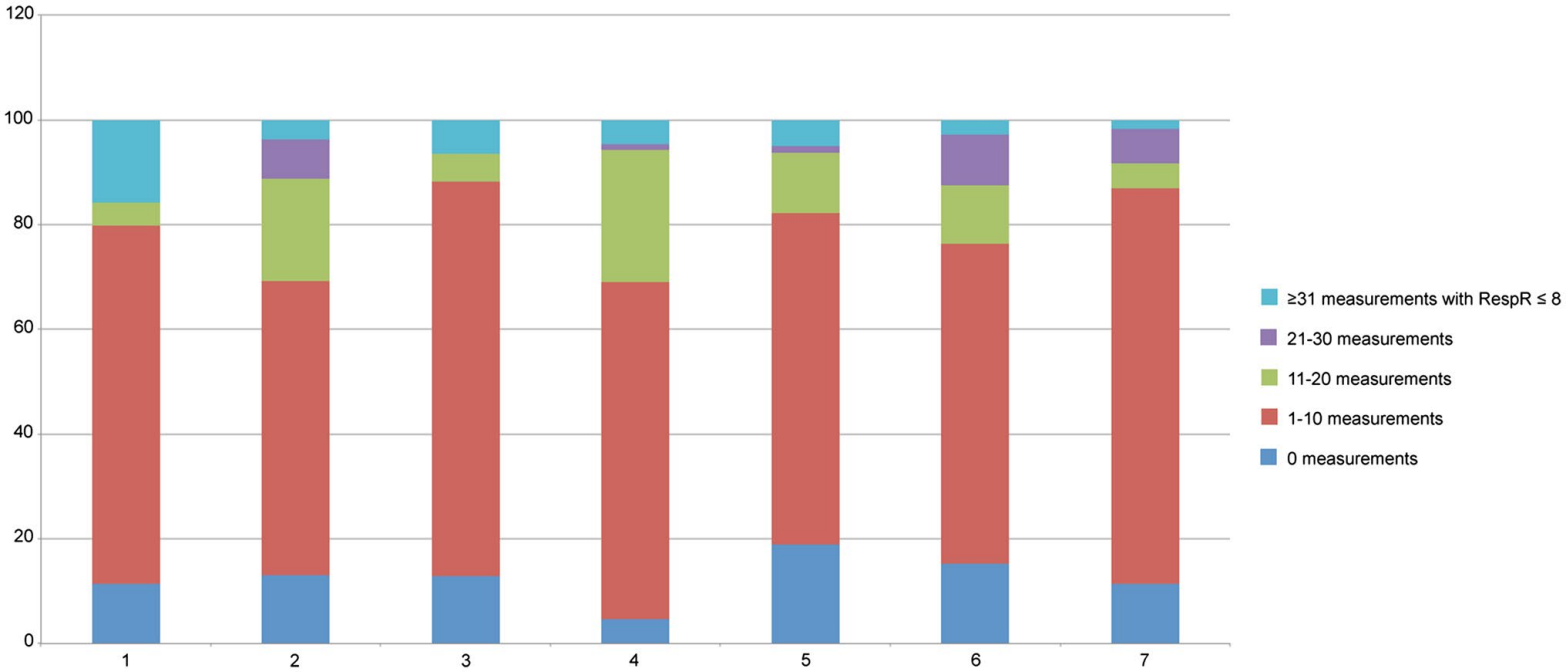
Percentage of patients with different numbers of measurements of RespR ≤ 8

Interestingly, bradypnea (RespR ≤ 8) was not uncommon and occurred during the entire hospital stay (Fig. 4). During the first post-operative night, 89% of patients experienced at least once a bradypnea. This was also observed in 81–95% of patients in the following time periods (day 2–night 4).

During day-time, in 3.9% of all valid measurements a RespR of ≤ 8 was noted, during night-time this was the case in 3.5% of all valid measurements. Remarkably, in 18 out of 119 patients (15%) a bradypnea was measured at least 30 times during the first post-operative night (equal to total duration equal or longer than 60 min).

Tachypnea with RespR ≥ 21 (sepsis criteria), or RespR ≥ 31 (local MEWS criteria) were detected 27,151 (16% of valid measurements) and 1009 times (0.6% of valid measurements), respectively.

**Fig. 5:**
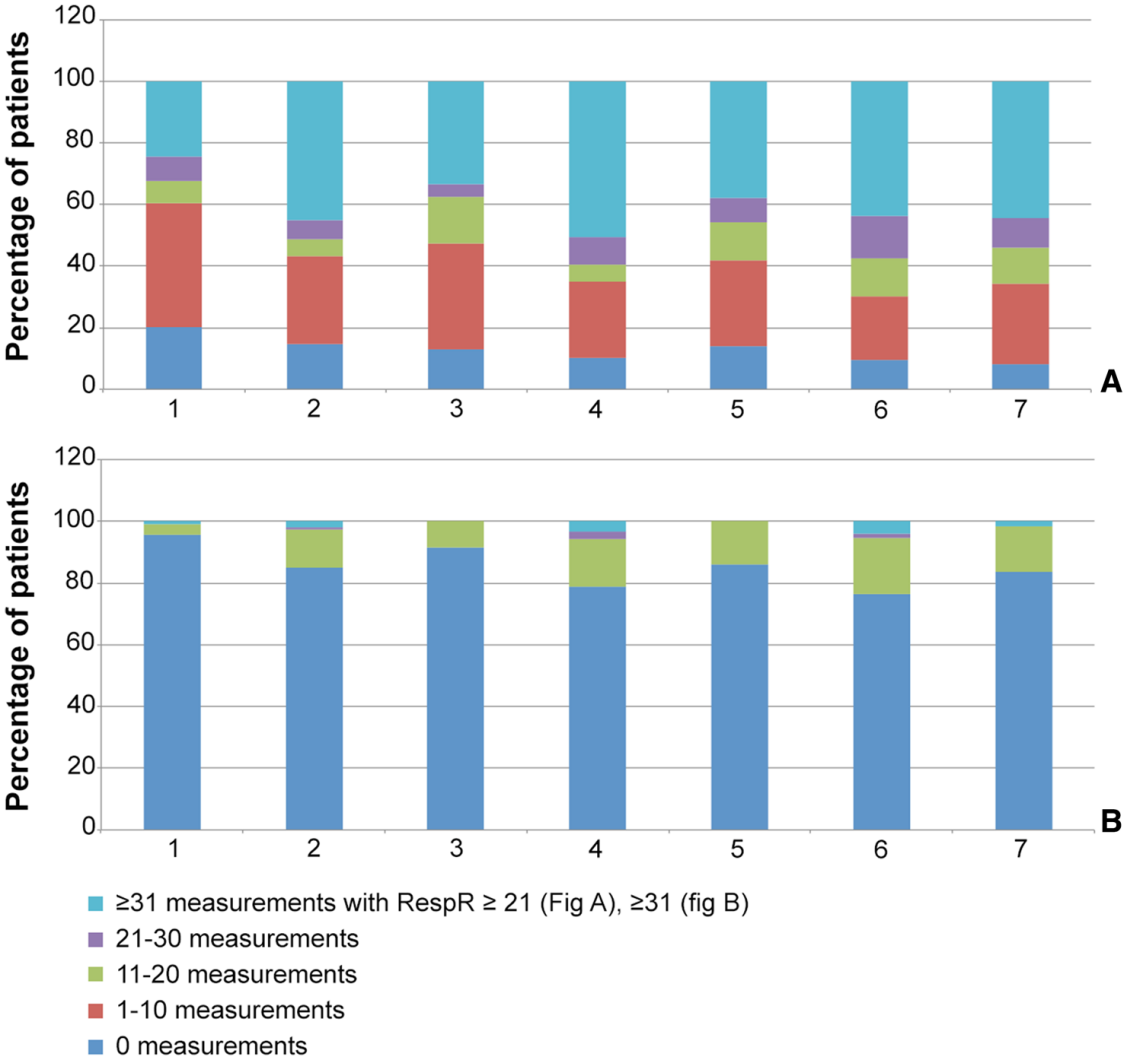
Percentage of patients with different numbers of measurements of RespR ≥ 21 or RespR ≥ 31

A RespR ≥ 21 occurred frequently during the entire hospital stay. At least one RespR ≥ 31 was observed in 10–15% of patients during the different time frames. Only in 0.4% of patients, RespR ≥ 31 was measured more than 30 times (total duration equal or longer than 60 min) during a predefined timeframe (see Fig. 5).

During hospital stay, one patient had a cutaneous reaction (red skin) to the ECG electrodes, and two patients showed skin irritations related to the patch; in these latter two patients the patch remained on the patient until discharge.

## Discussion

In this prospective observational study we demonstrated that it is feasible to measure electronically RespR for up to four post-operative days in a surgical ward using a wireless monitoring system, allowing for patients to normally mobilize during the postoperative period and not producing sleep deteriorations: 88% of included patients had more than 67% of valid RespR measurements. This was true in all timeframes, e.g., during day- and night-time, on all days.

As hypothesised, the RespR was lower than the previously reported value of 20: the median RespR was 15, with 50% of measurements detecting 13–19 breaths per minute. In our opinion this is a meaningful lower respiratory rate than normally determined by nurses. This observation might also have influences on the respiration value of MEWS systems. Our data are the first in a pure post-surgical cohort, and influences of perioperative pain therapy using opioids might have influenced our results leading to a lower RespR.

Interestingly, a clinically relevant number of RespR measurements in our patients showed brady- (RespR ≤ 8) or tachypnea (RespR ≥ 31).

In recent years, the industry has provided physicians and nurses with different techniques for continuously measuring vital parameters including respiratory rate on the ward [12, 25], but technical, organisational and financial arguments still withhold us from using these systems [26–28]. Most monitoring systems still use cables and may lead to unwanted immobilization of the respective patients [29]. Manually assessment of vital parameters might take up to 10 mins, meaning that even if vital functions are measured every 4 h (6 times a day), this will result in only 60 min of direct surveillance of the ward patient within 24 h, leaving the post-operative ward patient un-monitored for 96% of the time. Taking vital parameters during night might add burden because of sleep deterioration and its consequences in the hospitalised patient [30]. Continuous wireless monitoring can help to dramatically reduce the interval for assessing vital parameters without affecting mobilization of the patient, or disturbing sleep.

We used the SensiumVitals® patch and focussed on measurement of respiratory rates. This patch also measures heart rate and axillary temperature. Hernandez-Silveira et al. studied the accuracy of the SensiumVitals® patch by comparing the obtained patch data to those measured by IntelliVue MP30 Philips bedside monitoring [9]. The data from this previously published study showed that the patch measured both, heart rate and RespR reliably [9]. In distinct patient groups, the mean difference between the two monitors was one beat per minute for heart rate and less than one breath per minute for RespR. The patch measured heart rate 80% of time and RespR 50% of the time. RespR values were rejected more frequently because the measurement by impedance pneumography is quite sensitive to motion artefacts [9]. In our present study, after extensive teaching of staff to adequately place the SensiumVitals® patch, we achieved valid RespR measurements 75% of the time. In line with previous data [9] we had more valuable data during the night-time, when patients are less mobile. A recent study exploring a similar patch type showed less accuracy of RespR between a wireless patch and a reference monitor [12]. However, the measurements with the patch showed much less intra-individual variability than measurements with the standard monitor, and thus it is possible that the reduced inaccuracy was due to the variability of RespR measurement by the reference monitor.

In the present study, in 8% of included patients the respiratory signal was rejected in more than 50% of measurements, and there were two patients with rejection of 72% or 80% of measurements (thus only 28% and 20% of valid measurements), respectively. This loss of monitoring signal might be less than re-assuring, as the clinician cannot predict which patients are likely to have respiratory rate disturbances. However, the SensiumVitals® patch determines RespR every 2 mins, resulting in a maximum of 30 measurements per hour and consequently a maximum of 24 × 30 = 720 measurements per day. Even with only 20% of valid RespR measurements (the least measurements observed in one of our patients), one will still obtain 144 measurements for RespR, contrasting the only six values if nurses obtain vitals manually every 4 h. Improvement of technology should focus on making the systems used for continuous wireless monitoring even more reliable.

As with other non-invasive techniques [31], the SensiumVitals® patch might be less useful in patients with distinct co-morbidities, like cardiac rhythm disturbances and implanted cardiac devices, as well as in obese patients [9, 32], although heart rate determination might be more affected in these circumstances than detection of RespR. Number of co-morbidities in our study population was low (Table 1), thus feasibility of respiratory rate measurement with the used wireless system in a cohort of patients with significant co-morbidities including respiratory and cardiovascular deviations should be performed in the future.

Several cardiopulmonary diseases, metabolic changes (e.g., acid–base status or glycaemic dysregulation), as well as adverse drug effects may directly affect RespR. About 80% of clinical staff—physicians and nurses—think that respiratory rate is a very good indicator for severe patient deterioration [33]. However, measurement of respiratory function (RespR or peripheral oxygen saturation) outside a high-dependency unit (intensive or medium care, postoperative recovery unit, operation room etc.) is not common and frequently performed inadequately [3, 19, 20]. Compared to standard registration of RespR by counting 1 min of breathing, nurses only counted correct values in 3% of measurements if the actual respiratory rate of the patient was below 12/min. In contrast, nurses measured correct in 76% of times for respiratory values of 18–22 [34]. These data underline the necessity to improve RespR measurement namely in those patients not breathing normally. In accordance with a recently published study in 67 post-surgical patients [35], our data show a median RespR of 15 (mean = 16). However, this is in contrast to previous publications showing a mean RespR of 18–20 when measured by an electronic monitoring system [13, 14], or a RespR of 16–25 when determined using a standardised method by a research team [15, 34].

In the present study, 89% of patients experienced bradypnea at least once during the first post-operative night, and bradypnea was also observed in 81–95% of patients in the following time periods (day 2–night 4). In 15% of patients, total time for bradypnea summed up to more than 60 min during the first night. Bradypnea might occur due to reduced respiratory muscle function caused by surgical incision [18], prolonged anaesthesia effects, and postoperative pain treatment with, e.g., opioids. Effective pain treatment using patient-controlled analgesia with opioids might compromise respiratory function leading to bradypnea [16]. It has been advocated that “no patient should be harmed by opioid-induced respiratory depression” [36], and continuous wireless monitoring might be one option to further improve patient surveillance.

In the early postoperative period patients might also suffer from tachypnea (RespR ≥ 31) due to e.g., hypovolemia, uncontrolled pain relief, metabolic disturbances, developing infection or cardiopulmonary co-morbidity. Measurements of RespR ≥ 31 were rare (0.6% of valid measurements) in our study, and as expected there were more episodes of tachypnea measured during the later post-operative days, supporting the current knowledge that patient deterioration namely occurs on post-operative days 2 to 5 [37, 38].

This study has also some limitations. The SensiumVitals® patch uses impedance pneumography to measure RespR. There is an ongoing debate whether impedance pneumography is capable of detecting obstructive apnea, since it measures a change in chest expansion and not actual respiratory flow [39, 40]. Some authors argue that obstructive apnea is not accurately measured by impedance pneumography, because the chest wall might continue to move during apnea [40]. In contrast, other authors argue that changes in impedance is almost linear to changes in lung volume, and is indeed able to detect obstructive apnea [39]. However, whether SensiumVitals® measures RespR accurately in patients with obstructive apnea has yet to be determined. Next to impedance pneumography used in the SensiumVitals® patch, there are other technological solutions using different methods of measuring respiratory rate which might have opportunities in different clinical settings [12, 25, 39, 41–44]. Which of these techniques best determines the correct RespR has not yet been investigated. We did not simultaneously measure reference values, and therefore we can not prove that the measured values resemble the real RespR. However, it has been shown previously that the SensiumVitals® patch reliably measured RespR when compared to a standard approach (e.g., counting RespR for a random 60 s interval during a 5 min period in a quietly breathing patient who is blinded to measurement period) [32]. In addition, the new wireless systems might be even more accurate than currently used reference methods, making prudent interpretation of received data necessary [12]. Notifications of incorrect measurements may result in false alarms, increasing workload of nurses and finally leading to alarm fatigue [45], with subsequent failure of the whole response system [46]. Further research should therefore focus on the best method to measure accurately RespR in postoperative patients most likely treated with opioids, and how to best prevent false alarms without loosing reliability for real deteriorations.

A recent systematic review with meta-analysis did not prove effectiveness of continuous or intermittent vital signs monitoring in preventing adverse events on general wards [47]. However, the number and quality of studies was too low to allow final conclusions. Recent publications support the need for continuous ward monitoring [48, 49], and several different systems are available, including new measurement techniques [50, 51]. In the current study we demonstrated that with the used wireless monitoring system it is feasible to electronically measure RespR up to 4 days post-operatively on the ward. Because changes of respiratory rate are the key component of deterioration, improving quality and number of measurements will most likely serve to improve outcome of these patients. However, this still has to be proven in an adequately powered outcome study.
